# The process-disruption hypothesis: how spelling and typing skill affects written composition process and product

**DOI:** 10.1007/s00426-021-01625-z

**Published:** 2022-01-08

**Authors:** Vibeke Rønneberg, Mark Torrance, Per Henning Uppstad, Christer Johansson

**Affiliations:** 1 University of Stavanger, Stavanger, Norway; 2 Nottingham Trent University, Nottingham, UK; 3 University of Bergen, Bergen, Norway

## Abstract

This study investigates the possibility that lack of fluency in spelling and/or typing disrupts writing processes in such a way as to cause damage to the substance (content and structure) of the resulting text. 101 children (mean age 11 years 10 months), writing in a relatively shallow orthography (Norwegian), composed argumentative essays using a simple text editor that provided accurate timing for each keystroke. Production fluency was assessed in terms of both within-word and word-initial interkey intervals and pause counts. We also assessed the substantive quality of completed texts. Students also performed tasks in which we recorded time to pressing keyboard keys in response to spoken letter names (a keyboard knowledge measure), response time and interkey intervals when spelling single, spoken words (spelling fluency), and interkey intervals when typing a simple sentence from memory (transcription fluency). Analysis by piecewise structural equation modelling gave clear evidence that all three of these measures predict fluency when composing full text. Students with longer mid-word interkey intervals when composing full text tended to produce texts with slightly weaker theme development. However, we found no other effects of composition fluency measures on measures of the substantive quality of the completed text. Our findings did not, therefore, provide support for the process-disruption hypothesis, at least in the context of upper-primary students writing in a shallow orthography.

## Introduction

Written composition—generating ideas and shaping them into coherent and accurate text—involves a sequence of processes, starting with content retrieval and structuring, and progressing through syntactic planning and spelling retrieval, to the motor planning and execution of keypresses or pen strokes. These processes can be thought of as forming a cascade (Olive, 2014; Roux et al., 2013; van Galen, 1991): processing at a specific level starts as soon as it is provided with input from the preceding (upstream) process. The upstream process is then freed to take new input, processing this in parallel with processing at other levels. It is this “just-in-time” processing (Christiansen & Chater, 2016) that allows competent writers (communicating non-demanding content) to write fluently with only rare hesitations. Difficulty at any level of this cascade of processes, however, forms a bottleneck that results in production disfluency: the writer’s typing (or pen movement) slows or pauses. Bottlenecks of this nature are more likely to occur when the writer lacks practice in one or more of the component mechanisms (see, for example, Ruthruff et al., 2001). This will be the case, for example, for adults writing in a language in which they are not fluent (e.g. Chukharev-Hudilainen et al., 2019) or—the focus of the present study—when the writer is a primary school student.

From a communicational point of view, disfluency in written production is not a problem. Unlike in speech, hesitation during written production, in most contexts, has no effect on readers’ understanding of what is written. Pauses that occur as a result of a child thinking carefully about content are, in fact, welcome. However, unexpectedly long pauses in particular at mid-sentence or within-word locations might be disruptive. Such disfluencies may result from bottlenecks at lower levels in the cascade, as children attempt to retrieve spelling and/or plan the motor actions necessary to form letters on the page or computer screen. Where these lower level transcription processes are the source of disfluency, this may have negative consequences for the quality of the resulting text. If for example a child struggles to spell a word, even if they finally retrieve the correct spelling, this processing difficulty may have consequences for more global features of the text. Fluent output is of course important in any context where the writing task is time constrained. Slower writing will, in many contexts, mean a shorter final product. But there is also the possibility that disfluency in output has knock-on effects for upstream processes. Fluent writing means planning what to say next independently of and without interference from the processing needed to spell and to inscribe^1^ the words that are currently being written. Christiansen and Chater (2016) argue that there is a fundamental “now-or-never” bottleneck in human language processing. In the context of written production, this means that delays associated with spelling or with inscription may disrupt the processes responsible for generating and structuring what to say next, i.e. there seems to be important timing issues for optimal text production.

Researchers exploring writing development have frequently suggested that competition for processing resources, in some form, links word-level difficulty with an inability to construct text that is informationally rich and coherently structured (e.g. Berninger et al., 2002; McCutchen, 1996; Torrance & Galbraith, 2006). This has been advanced as an explanation for, for example, the finding that the compositional quality of students’ writing improves as a result of training in handwriting (Alves et al., 2016) and typing (van Weerdenburg et al., 2019). The relationship between writers’ transcription skill (skill in spelling and in inscription) and the compositional quality of their text has been explored more directly in a number of studies (e.g. Abbott et al., 2010; Alves & Limpo, 2015; Connelly et al., 2005; Graham et al., 1997; Jones & Christensen, 1999; Kim et al., 2015). In a meta-analysis of studies with children from kindergarten to 12th grade, Kent and Wensek (2016) found a mean standardized effect of 0.49 for the relationship between spelling-test performance and writing quality (18 effects) and also of 0.49 for the effect of handwriting fluency (17 effects). Feng et al. (2019) in an overlapping meta-analysis (9 effects) found a mean effect of 0.41 of handwriting fluency on, specifically, substantive quality (i.e. quality of just the content and structure of the completed text, ignoring spelling and handwriting accuracy). Studies have also explored transcription effects on written productivity—the number of words of spontaneous text composed per minute or within a fixed time limit. For example, Graham et al. (1997) found that productivity in English-speaking upper-primary school students was predicted by accuracy on a spelling-to-dictation test and speed of written alphabet recall. Alves and Limpo (2015) found similar effects for students writing in Portuguese, a language with a much more transparent orthography (i.e. with more regular sound-to-letter correspondence). Feng et al. (2019) who found an average effect of handwriting fluency on productivity of 0.53 (12 effects), averaging across a small number of studies, drew similar conclusions. It is worth noting that in most cases the measure attributed to handwriting fluency in all of these studies was speed of handwritten alphabet recall, a measure that includes a memory component and alphabet knowledge in addition to the ability to fluently move the hand.

One possible explanation for these effects is the “process-disruption hypothesis”: Weaker spelling and inscription skills result in production disfluency and this disrupts the writing process in ways that result in damage to text quality. With regards spelling effects, however, there are two reasons why the deficit associated with inaccuracy in spelling-to-dictation may not necessarily mean that the child’s writing processes will be disrupted when they compose full text. First, inaccuracy is not necessarily associated with disfluency. As long as a word is retrieved and produced fluently this will not disrupt upstream processes, regardless of whether it is spelt correctly. Torrance, Rønneberg, et al. (2016) for example, found that words wrongly spelled in upper secondary students’ written compositions, after statistical control for length and frequency, were produced more, rather than less fluently than words that were spelled correctly. Second, accurately spelled words are not necessarily produced fluently. Fluent spelling is much more likely to be achieved when orthographic retrieval is by a direct (lexical) route (e.g. Delattre et al., 2006; Martin & Barry, 2012). However, when writing in a transparent orthography as was the case in the present study, assembling the spelling of a word by phoneme–grapheme translation will typically be successful. Children who do not have the lexical knowledge required to make this direct route possible will therefore nearly always be accurate, but are more likely to be slow. For these reasons, association between children’s accuracy on a spelling task and the quality of their written composition is, at best, only weak evidence that difficulty with spelling disrupts upstream (higher-level) cognitive processing. Child-level factors that independently explain both spelling ability and composition performance probably provide a more parsimonious explanation.

Strong evidence that difficulty with spelling retrieval and/or inscription disrupts higher-level processing requires demonstrating two effects: first, that ability to spell fluently and to inscribe fluently, measured in tasks where these abilities are clearly isolated from other language and literacy-related abilities, predicts word-level fluency when composing full text; second, that word-level fluency when composing a text in turn predicts the compositional quality of the final product.

Word-level fluency here refers to two specific phenomena: the time to initiate the production of a mid-sentence word,^2^ and the time to initiate production of each letter within a word. In typewriting these can be measured as the time between releasing the spacebar and pressing the character at the start of a new word—henceforth *word-initial latency*—or the time between keypresses within a word (*within-word latency*). In competent adult writers, word-initial latencies are typically between 250 and 400 ms, and within-word latencies are in the region of 150–250 ms (Chukharev-Hudilainen et al., 2019; Conijn et al., 2019). The mental processing underlying word-initial latency and within-word latency is different, but with some overlap. Pre-word processing must include lexical retrieval. In written picture word naming task, performed by adults, frequency affects word-initial latency across a range of languages including Norwegian—the language of the present study—but does appear to affect within-word latencies (Torrance et al., 2018). Pre-word lexical retrieval includes spelling retrieval—difficult-to-spell words are associated with longer response times in spelling to dictation (e.g. Delattre et al., 2006). There is some evidence however, that spelling processing is not always complete at inscription onset: within-word latencies also increase for more difficult words, a finding that appears to hold true in both deep and more transparent orthographies and for adults and children (Bonin et al., 2012; Delattre et al., 2006; Rønneberg & Torrance, 2019; Torrance et al., 2018). Both word-initial and within-word latencies will, of course, also be associated with the motor planning and execution of the keystroke.

Interpretation of these latencies when the writers produce continuous text rather than isolated words is complicated, however, by the fact that at least some of the processing necessary to determine the next keystroke can be conducted in parallel with the output of previous letters or words. This is a direct consequence of the cascading nature of the text production process. So all processing associated with lexical/orthographic retrieval of the next word, for example, does not necessarily occur in the interval after the terminating keystroke of the previous word. Typical means for word-initial and within-word latencies in fact obscure two different distributions of interkey intervals (Roeser et al., 2021), one associated with intervals dependent only on the time needed to motor plan and execute the next keystroke—processing that takes around 150 ms (Van Waes et al., 2021)—and a distribution of longer intervals that occur when the writer has to “pause” to engage in additional planning before the keystroke can be output. At a certain point, these longer intervals reach a threshold where they represent processing that is potentially disruptive—i.e. that has the potential to negatively affect the quality of the writer’s output.

A handful of studies have explored the effects of spelling and inscription ability on latency times during composition. In some of these studies latency times above a certain threshold, typically 2 s, are termed pauses. Alves and Limpo (2015) found that, in both lower and upper-primary school students composing text by hand, the mean number of words between pauses of more than 2 s (a measure referred to as “burst length”) was positively correlated with handwriting fluency (speed of written alphabet recall) and with accuracy in a spelling-to-dictation task (i.e. that students with better handwriting skills and spelling skills paused less, when controlling for the number of words written). Limpo and Alves (2017) report similar findings in a sample of just 2nd grade writers. Alves et al. (2016) found that training in handwriting reduces pausing in second grade writers, but that training in spelling does not. Torrance, Rønneberg, et al. (2016) found longer latencies at both word boundaries and within words, in adolescents with dyslexia (and therefore with weaker spelling skills) composing by keyboard compared to controls. In particular, these students were much more likely to pause within words for periods of 1–2 s, delays that might plausibly have a disruptive effect on upstream processes.

A similarly small number of studies have explored the relationship between pausing during production and the quality of the final text. Grewal and Williams (2018), in a sample of school age children writing in English as a second or other language, examined effects of mean burst length (with a 2 s pause threshold) and of count and summed duration of pauses over 250 ms on features of the final product, controlling for various child-level factors. They found positive correlations with spelling accuracy and lexical richness, but no relationship with a composite text quality measure. Alves and Limpo (2015) found that both burst length and mean duration of pauses, again with a 2 s threshold, predicted holistic ratings across grades 2–7 in children writing by hand in Portuguese. The effects were relatively weak after the fourth grade and absent at the sixth grade when children wrote narratives. Asker-Árnason et al., (2010, 2012), in a sample of mainly of secondary school-age students typing narratives, found no relationship between proportion of time on task spent in pauses of greater than 2 s and narrative quality. Deane (2014) found significant and non-trivial effects of typing fluency on text quality, after control for various product characteristics including spelling accuracy, in a large sample of eighth grade students. The composite nature of the writing fluency measures that they report, however, which aggregated measures including burst length and interkey interval (both subject means and SDs) and deletion counts, makes interpretation difficult. Studies with adult writers have found fewer effects. Medimorec and Risko (2017) found evidence of a slight tendency for students who pause more before words to write shorter sentences, but few other effects. Conijn (2020), in a sample of university students writing (proficiently) in English as a second language, found no correlation between subjective text ratings and either word-initial or within-word latencies, or any of a large number of other pause-related measures.

### The present study

The aim of our study was to provide a strong test of the hypothesis that difficulty with spelling and/or with inscription disrupts the processing necessary to produce text that is compositionally well formed. Our study sampled sixth grade students writing by typing in a relatively shallow orthography (Norwegian). In this context, we examined the putative causal chain from spelling and inscription ability to production fluency, and from production fluency to the compositional quality of the completed texts. We built on previous research in the following ways. First, our measures of spelling and inscription ability focussed specifically on relevant measures of output fluency, rather than accuracy. For reasons that we have discussed, this is particularly important for assessment of spelling ability. As in previous studies, we assessed spelling ability with a spelling to dictation task, but focussed on speed of retrieval and production rather than accuracy. Second, our analysis of latency times took into account whether these occurred within or before words—the locations likely to be associated with transcription difficulties—or at higher-order text locations. Third, we bring these measures together in a single study that looks at both ability effects on process and process effects on product. For reasons that we have discussed, we believe that these three features are necessary to provide more direct evidence of the process-disruption hypothesis than is, to our knowledge, provided in the existing literature.

Our decision to study typed production in sixth grade students was both expedient and principled. Norwegian children, as in most educational contexts, typically learn to handwrite before they learn to type. There are, however, national curriculum expectations that students achieve a reasonable level of typing proficiency by the time they leave primary school (Ministry of Education and Research, 2019). As early as third grade, children therefore typically have sufficient typing fluency to create multi-sentence compositions within a reasonable time limit (see, for example, von Koss Torkildsen et al., 2016), although we anticipated greater variability in skills than might be expected from, for example, a current young adult sample. This, combined with the ease with which writing time course measures can be extracted from typed production, motivated the choice of typing as output medium in our study.

## Method

### Design and participants

Norwegian sixth grade students typed argumentative texts using a simple text editor that provided accurate timing for each keystroke. They also completed a timed key-finding task designed to measure keyboard knowledge, a timed spelling-to-dictation task, and a typing fluency task involving writing a familiar sentence repeatedly from memory.

The sample comprised 101 students (61 female) with a mean age of 11 years and 10 months. Participants were recruited from seven classes across four different suburban public schools. Students who reported that they did not speak Norwegian at home, who were identified by the teacher as having serious behavioural challenges, or who were absent for one or more of the testing sessions were excluded from the sample.

### Measures

#### Written composition

Participants were randomly allocated one of three composition topics (e.g. *You are a scientist, and you have built a time machine that actually works, but you can only use it once. Think carefully. To what time in the past or in the future would you go? Give reasons for your choice.*) The writing topics were introduced in the classroom and students took part in a brainstorming session before starting to write. Students were then given 25 min to complete their text, and they were instructed to write under “exam conditions”. They were informed that the person conducting the tests together with a colleague would read their texts.

#### Fluency (process) measures

**Fig. A1:**
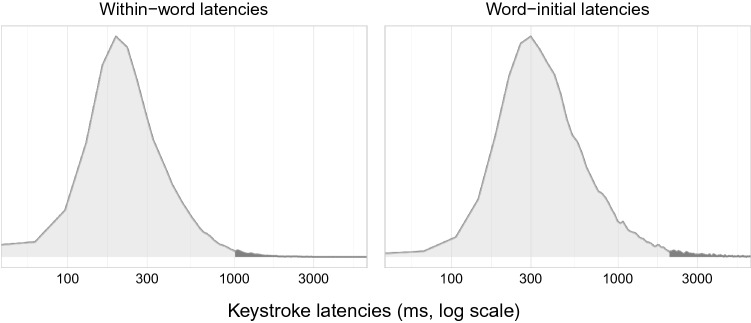
Distributions (Gaussian kernel density estimates) of keystroke latencies (interkeystroke intervals). The dark region in the positive tail represents latencies identified in our analyses as disfluencies (mid-word, > 1 s; word-initial, > 2 s)

All keystrokes were recorded using custom software implemented within and taking timing accuracy from the SR Research Experiment Builder programming environment (SR Research Experiment Builder, 2017; Wengelin et al., 2009). We identified interkey intervals (the time between pressing one key and the next) at three different locations within the text: *within-word latency*, defined as the interval between any two character keys within a word; *word-initial latency*, defined as the interval between pressing the space key and the first character of a new (non-sentence-initial) word; and *sentence-initial latency*, the word-initial interval for a word at start of an orthographically marked sentence (i.e. a word preceded by sentence-terminating punctuation and one or more spaces).^3^ In addition to interkey interval times at these locations, we also identified whether or not the interval exceeded specific threshold (i.e. was a “pause”) and therefore represented a disfluency in the writer’s production. This captures the idea that processing at a specific location in the text will only be disruptive to higher level processing if it takes substantially longer than is typical for that location. We identified a *within-word disfluency* if the interval exceeded 1 s and a *word-initial disfluency* if the interval exceeded 2 s. 2 s has been treated as the threshold at which an interkey interval becomes a “pause” by convention (Strömqvist et al., 2006) and in several, but not all, of the studies that we cited in our introduction. The modal word-initial latency for children in our sample composing continuous text was in the region of 300 ms (See Appendix Fig. A1). Norwegian adult writers are able to apperceive, retrieve and start typing the name of a pictured object in around 1300 ms (Torrance et al., 2018). Given these values we believe that 2 s represents a word-initial latency that might safely be assumed to indicate additional or different processing from that associated with typical, fluent production. Modal within-word latency in our sample was, as might be expected, considerably lower at just under 200 ms (Appendix Fig. A1) and the distribution had a shorter tail. We therefore adopted a lower threshold when identifying within-word disfluencies. As can be seen from Fig. A1, the 2s and 1s thresholds delimited a roughly comparable proportion of the positive tail of their respective distributions.

#### Text quality measures

Prior to quality rating, all texts were corrected for spelling, but punctuation and grammatical errors were preserved. Holistic (reader-based) measures of text quality were adapted from the assessment criteria for the WIAT-II UK essay task (Wechsler, 2006). Texts were scored for organization, theme development, and overall quality. *Organization* (1–17 points) was awarded for the presence of a topic sentence, sentence order, text macro-structure, and signposting. *Theme development* (1 to 8) assessed the argumentation, with points awarded for maintaining focus, a clear position statement, and the quality of argument and counterargument. O*verall quality* (1 to 6) has scores representing how well the essay implied a position, whether the text gave reasons for its position, and the clarity, organization, detail and logic of its message, and the general quality of the written language. Grammatical accuracy was not considered. All texts were rated independently by two trained raters, with good agreement: Cohen’s weighted kappa; 0.85 for organization, 0.82 for theme development, and 0.87 for overall quality.

We also recorded *text length*, in words, number of *spelling errors*, and *lexical diversity*. Lexical diversity was measured just for content (non-function) words using McCarthy's (2005) measure of textual lexical diversity (MTLD) statistic. Unlike type–token ratio, this is independent of text length (*r* = 0.07 in our data) and offers better sensitivity than comparable measures (Torruella & Capsada, 2013). Correlation with type–token ratio in our data was 0.44.

#### Spelling fluency

Participants completed a computer-administered spelling-to-dictation task consisting of 32 items that formed a standardized Norwegian spelling test (Skaathun, 2013). Items comprised a mixture of words with straightforward phoneme–grapheme mapping and words with a spelling challenge (reported in Rønneberg and Torrance, 2019). Spelling challenges included consonant doubling, consonant clusters, words including phonemes that are hard to differentiate and silent letters. Words varied in length, the shortest word consisted of two letters and the longest words consisted of ten letters. Participants first heard the target word presented in a sentence, with position in the sentence varying, and then heard the target word. They were instructed to then type the word as quickly and accurately as possible. The task was implemented within SR Research Experiment Builder, with keystrokes captured using the same custom code as used for the composition task.

We extracted two spelling fluency measures. *Spelling response time* was timed from onset of the target word to time of first keypress. Note that response time in this context provides a measure of spelling fluency that was largely independent of typing skill. *Spelling within-word latencies* represented the interkey intervals once typing had commenced (aggregated by taking the median for analyses in which spelling fluency was a predictor variable).

#### Typing fluency

Typing fluency, in the present context, measures the time it takes children to type familiar words with known and practiced spelling and with a normal-range digraph and letter frequency. Our task therefore differed from tasks that involve alphabet recall or reading and copying unfamiliar sentences that have typically been used in previous research (e.g. Barnett et al., 2007; Graham et al., 1997). Before sitting down at individual computers, students were asked to memorize these two sentences: *Jeg gleder meg til bursdagen min. Da får jeg en fin gave.* (I am looking forward to my birthday. I am going to get a nice present.) These were written on a blackboard in front of the class and children spoke them in unison three times. They then saw the sentences on their computer screen and were asked to copy them five times. The sentences were then removed and students were asked to type them as many times as possible within 1 min. We extracted two typing fluency measures from this final, speeded typing task: *median word-initial latency* (interval between pressing the space bar and the word-initial key, excluding the two sentence-initial words) and *median within-word latency*. Again, the task was implemented within SR Research Experiment Builder, with keystrokes captured using the same custom code as used for the composition task.

#### Key-finding ability

The key-finding task was intended as a measure of procedural keyboarding knowledge independent of other translating processes. Students heard letter names and were required to press the corresponding key as quickly as possible. Letter names were pre-recorded, and students heard these through headphones. They completed 28 trials comprising 14 different letters, each repeated twice, in random order but avoiding the same letter being presented consecutively. This task gave one measure—*keyfinding speed*—calculated as the mean response time across all trials with a correct response. The mean proportion of correct responses was 0.89 (SD = 0.07). The task was implemented within the SR Research Experiment Builder.

#### General ability

We also included two general ability measures—*non-verbal reasoning* and *reading comprehension*—as covariates in our statistical models. Students completed Raven’s Standard Progressive Matrices task (Raven, 1981) as a measure of general non-verbal cognitive ability. We accessed students reading comprehension scores from a national, standardized task. Students read a total of five texts and then answered questions 29 (mainly multiple-choice questions, but three questions that required students to write) designed to assess their understanding of what they had read. Scores were missing for 17 students for this task. These were replaced by the mean.

### Procedure

Testing took place over four sessions on consecutive days between mid-February and April in the students’ sixth grade year. With the exception of the national reading tests, all tasks were administered by the first author and a trained research assistant.

## Results

We first examine the effects of keyboard knowledge, spelling ability and typing fluency on fluency when composing text, and then the relationship between these composition fluency measures and the quality of the completed text.

### Composition fluency

**Fig. 1:**
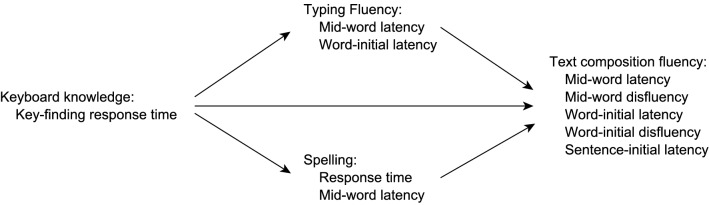
Path model for predictors of composition fluency

We hypothesized the possible causal relationships among measures of children’s spelling and typing ability and their fluency when composing text as detailed in Fig. 1. The fluency of text composition results directly from the ability to spell and type fluently. There is also the possibility of a direct relationship between keyboard knowledge—measured by key-finding response time—and composition fluency, although this effect might be mediated entirely by typing fluency. Typing fluency and spelling fluency—as assessed in this study—are dependent on keyboard knowledge.

We evaluated piecewise structural equation models (Shipley, 2003, 2009, 2013) with separate models for each of the five composition fluency measures. This approach involves evaluating separate regression models for each dependent variable within the overall path model. Goodness of fit for the path model as a whole is evaluated with a test of directional separation (*d-sep*; Shipley, 2003). The d-sep test entails evaluating all paths that are not hypothesized by the path model and then using *p* values from these models to calculate Fisher’s *C* statistic (Fisher, 1954). If Fisher’s C gives *p* > 0.05, this is evidence against paths absent from the model providing information (i.e. evidence that the hypothesized path model provides good fit). AIC, calculated from Fisher’s *C* with a penalty for the total number of parameters estimated, can then be used to compare different path models (Shipley, 2013). To avoid overfitting, we used the corrected version AICc. A difference in AICc of greater than 4 between a candidate model and a competitor can be considered moderate evidence for preferring the candidate model, and of greater than 10 as strong evidence (Burnham & Anderson, 2004). Our use of this piecewise approach allowed us to break down the overall path model into individual regression models with varying random effects specifications.

Keystroke data from the composition task and typing fluency tasks comprised multiple observations (one per keystroke). These observations were clustered within participant and within composition topic. Observations in the spelling fluency task data were also clustered within the item. We fitted mixed-effects regression models with, for all models, random by-participant intercepts. Models where composition measures were the outcome also included random intercepts for the three composition topics. Models where spelling fluency was the outcome also included by-item random intercepts. Statistical significance for individual parameters (path coefficients) was established either from *z* (for logistic regression models) or from *t* with the Satterthwaite approximation for denominator degrees of freedom (all other component models). Component models were implemented using the R lme4 package (Bates et al., 2015), and the piecewise structural equation modelling (evaluating the path model as a whole) made use of functions from piecewise SEM (Lefcheck, 2016).

For each of the five composition fluency outcome measures, we compared the full path model, as shown in Fig. 1 with models that excluded paths from predictor variables. For path models predicting composition within-word latency and disfluency, we used within-word latencies as our measures of typing fluency and of spelling fluency, and for models predicting composition word-initial latency, disfluency and sentence-initial latency we used word-initial latency as our measure of typing fluency and response time as our measure of spelling fluency. For component models in which these measures were predictors, we aggregated within participants, taking the median value. All latency and response time measures were trimmed within subject and text location at ± 2.5 SD and then log-transformed. For component models that predicted disfluency (probability of pausing), whether or not specific interkey interval threshold exceeded the pause threshold was represented by a binary dummy variable, and we modelled with logistic mixed-effects regression.

All component models included reading comprehension and non-verbal reasoning measures as control for general ability effects. There was evidence that these factors explained variance in word-initial latency during composition, in key-finding response time, and in within-word latency in the typing fluency task (*χ*^2^(2) = 6.37, *p* = 0.041; *χ*^2^(2) = 6.47, *p* = 0.039; *χ*^2^(2) = 10.76, *p* = 0.005, respectively, relatively to an intercept-only model). We did not find evidence of an effect on other variables (*χ*^2^(2) ≤ 4.50, *p* > 0.05 in all cases).

#### Findings

**Fig. 2:**
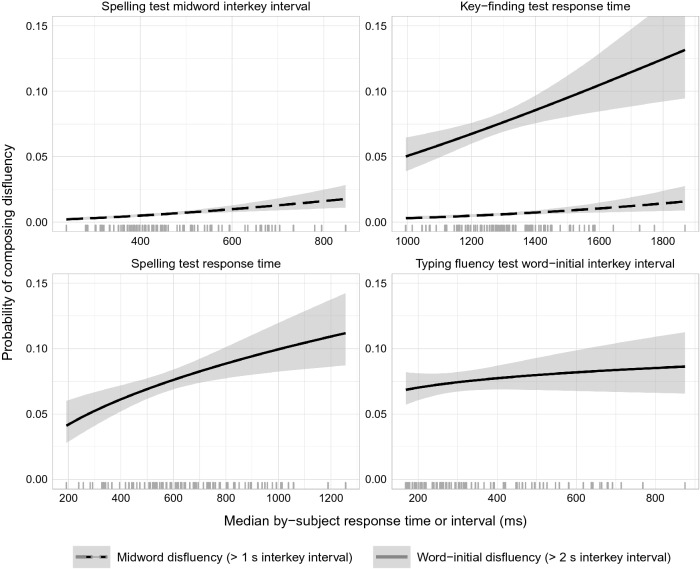
Estimated effects of typing and spelling fluency measures on the probability that an interkey interval is sufficiently long to be considered a disfluency, with 95% CI, showing just those effects appearing in best-fit models

Table 1 reports means for and bivariate correlations among composition process measures and the various ability measures. Distributions for composition fluency measures are given in the Appendix. Model fits and comparisons are shown in Table 2. Standardized path coefficients from the best-fit model are shown in Table 3 and illustrated in Fig. 2. Key-finding response time predicted both typing and spelling fluency in all best-fist models.

**Table 1:** Transcription ability and composition process measures: descriptive statistics and bivariate correlation

	M (SD)	Word-initial disfl.	Mid-word disfl.	Mid-word latency	Word-initial latency	Sentence-initial latency	Typing mid-word	Typing word-initial	Key finding	Spelling RT	Spelling latency	Reading
Composing text												
Proportion word-initial disfluency	0.094 (0.09)											
Proportion mid-word disfluency	0.051 (0.07)	0.83										
Median mid-word latency	323 (128)	0.63	0.80									
Median word-initial latency	596 (313)	0.76	0.77	0.74								
Median sentence-initial latency	2103 (1882)	0.45	0.41	0.13	0.11							
Typing fluency												
Median mid-word latency	310 (139)	0.33	0.44	0.71	0.61	0.25						
Median word-initial latency	371 (161)	0.33	0.42	0.58	0.44	0.28	0.46					
Key-finding												
Median response time	1336 (179)	0.55	0.60	0.65	0.56	0.32	0.41	0.36				
Spelling												
Median response time	768 (459)	0.60	0.57	0.61	0.61	0.36	0.56	0.36	0.46			
Median mid-word latency	493 (163)	0.66	0.57	0.82	0.68	0.44	0.60	0.44	0.52	0.69		
General ability controls												
Reading comprehension	22.7 (5.8)	− 0.11	− 0.24	− 0.12	− 0.10	− 0.02	− 0.06	− 0.15	− 0.18	− 0.36	− 0.39	
Non-verbal reasoning (Raven)	42.2 (5.4)	− 0.06	− 0.19	− 0.06	− 0.06	− 0.08	− 0.11	0.02	− 0.25	− 0.13	0.00	0.22

For correlations, |r|> 0.31, *p* < 0.001; |r|> 0.23, *p* < 0.01. Latency and response times measures were aggregated within participants

**Table 2:** Composition fluency: model fits and comparison for the full path model (Fig. 1) and potential competitors

Model	C	df, *p*	AICc	ΔAICc
Word-initial latency				
All paths	3.52	29, 0.967	62.29	
Excluding path from spelling fluency	20.75	28, 0.023	77.36	15.07
Excluding path from typing fluency	20.12	28, 0.028	76.73	14.44
Excluding path from key-finding	20.10	28, 0.028	76.71	14.42
Excluding paths from spelling and typing fluency	37.36	27, 0.000	91.92	29.63
Word-initial disfluency				
All paths	3.52	28, 0.967	60.24	
Excluding path from spelling fluency	17.43	27, 0.065	72.10	11.86
Excluding path from typing fluency	6.20	27, 0.799	60.86	0.62
Excluding path from key-finding	18.42	27, 0.048	73.09	12.85
Excluding paths from spelling and typing fluency	20.11	26, 0.028	72.73	12.49
Mid-word latency				
All paths	24.68	29, 0.006	83.33	
Excluding path from spelling fluency	67.80	28, 0.000	124.40	41.07
Excluding path from typing fluency	40.74	28, 0.000	97.35	14.02
Excluding path from key-finding	35.42	28, 0.000	92.03	8.7
Excluding path from spelling and typing fluency	83.86	27, 0.000	138.42	55.09
Mid-word disfluency				
All paths	24.68	28, 0.006	97.96	0.41
Excluding path from spelling fluency	44.71	27, 0.000	114.63	17.08
Excluding path from typing fluency	27.63	27, 0.002	97.55	
Excluding path from key-finding	37.67	27, 0.000	107.59	10.04
Excluding path from spelling and typing fluency	47.66	26, 0.000	114.29	16.74
Sentence-initial latency				
All paths	3.52	29, 0.967	65.10	1.9
Excluding path from spelling fluency	4.28	28, 0.934	63.32	0.12
Excluding path from typing fluency	5.66	28, 0.843	64.94	1.74
Excluding path from key-finding	4.56	28, 0.919	63.72	0.52
Excluding paths from spelling and typing fluency	6.42	27, 0.778	63.20

**Table 3:** Predictors of composition fluency. Path coefficients and 95% CI from best-fit models

	Composition fluency measure				
	Word-initial latency	Word-initial disfluency	Mid-word latency	Mid-word disfluency	Sentence-initial latency
Key-finding → typing fluency	0.28 [0.16, 0.41]***	0.28 [0.16, 0.41]***	0.23 [0.13, 0.33]***	0.23 [0.13, 0.33]***	0.28 [0.16, 0.41]***
Key-finding → spelling fluency	0.22 [0.14, 0.30]***	0.22 [0.14, 0.30]***	0.34 [0.26, 0.43]***	0.34 [0.26, 0.43]***	0.22 [0.14, 0.30]***
Key-finding → composition fluency	0.14 [0.07, 0.21]***	0.22 [0.09, 0.35]***	0.09 [0.03, 0.15]**	0.36 [0.28, 0.70]***	0.13 [0.02, 0.23]*
Spelling fluency → composition fluency	0.16 [0.08, 0.24]***	0.25 [0.10, 0.39]***	0.24 [0.17, 0.31]***	0.49 [0.16, 0.55]***	–
Typing fluency → composition fluency	0.13 [0.07, 0.19]***	0.06 [− 0.05, 0.18]	0.12 [0.06, 0.18]***	–	–

Values are standardized effects. *p < 0.05, **p < 0.005, ***p < 0.001

Blank cells represent parameters that were absent in the best-fit model

Both *word-initial latency* and *within-word latency* during composition were best predicted by the full path model (Fig. 1). Spelling fluency was a relatively strong predictor in both cases. Both key-finding speed and typing fluency were also positive predictors but with somewhat weaker effects.

The best-fit models for both *word-initial disfluency* and *within-word disfluency* did not include statistically significant effects of typing fluency. Both key-finding speed and, in particular, spelling response time were relatively strong positive predictors. Effects on composing disfluency are illustrated in Fig. 2.

For *sentence-initial latency*, differences in fit among competing models were minimal. The best-fitting model included just a weak, but statistically significant, effect of key finding.

### Composition quality

Analyses reported in this section aimed to establish the association between composition fluency measures and the quality, accuracy and length of the resulting text. Data structure was straightforward, with one observation per participant for all variables. For each outcome variable, we fitted a series of five, nested, linear regression models. We started with a baseline (intercept-only) model (Model 0), then added effects for reading comprehension and non-verbal reasoning, as general ability controls (Model 1), then median within-word and word-initial latencies (Model 2), then proportion of within-word and word-initial disfluencies (pauses, Model 3), and finally median sentence-initial latency (Model 4). Model comparison was by likelihood ratio *χ*^2^ test.

#### Findings

Table 4 reports descriptive statistics for written product measures, and bivariate correlation with the composition fluency measures. Table 5 gives model comparison statistics from the incrementally fitted regression models. Table 6 gives parameter estimates from the best-fit model for each outcome variable.

**Table 4:** Descriptive statistics for length, lexical diversity (MTLD), spelling accuracy, and quality of completed texts. Means and bivariate correlations, including correlation with composition fluency predictors

	M (SD)	Length	Lexical diversity	Spelling errors	Thematic development	Organization	Overall quality
Completed text							
Length (words)	131.8 (51.5)						
Lexical diversity	71.3 (55.9)	0.07					
Spelling errors (proportion)	0.04 (0.05)	− 0.10	− 0.07				
Thematic development	5.23 (1.08)	0.21	0.00	− 0.21			
Organization	6.24 (2.32)	0.26	0.05	− 0.29	0.41		
Overall quality	2.69 (0.80)	0.48	0.04	− 0.27	0.52	0.72	
Composition fluency							
Median mid-word latency		− 0.28	− 0.07	0.33	− 0.37	− 0.14	− 0.23
Median word-initial latency		− 0.43	− 0.12	0.26	− 0.25	− 0.10	− 0-.23
Proportion mid-word disfluency		− 0.29	− 0.08	0.42	− 0.30	− 0.11	− 0.20
Proportion word-initial disfluency		− 0-.39	− 0.05	0.35	− 0-.29	− 0.20	− 0.29
Median sentence-initial latency		− 0.29	− 0.14	0.30	− 0.15	− 0.25	− 0.26

For correlations, |r|> 0.31, *p* < 0.001; |r|> 0.23, *p* < 0.01

**Table 5:** Comparison among models predicting text length, lexical diversity, spelling accuracy, and text quality; likelihood ratio χ^2^, degrees freedom, and *p*

Fixed factor(s) added	Length	Lexical diversity	Spelling errors	Thematic development	Organization	Overall quality
General ability: reading comprehension, non-verbal reasoning (Model 1)	(2.36, 2, 0.307)	(2.90, 2, 0.235)	7.23, 2, 0.027	8.41, 2, 0.015	10.57, 2, 0.005	13.24, 2, 0.001
Word-level latencies: median mid-word latency, median word-initial latency (Model 2)	21.60, 2, < 0.001	(1.22, 2, 0.545)	12.04, 2, 0.002	14.09, 2, 0.001	(1.67, 2, 0.434)	6.00, 2, 0.050
Word Disfluencies: proportion word-initial disfluency, proportion mid-word disfluency (Model 3)	(2.47, 2, 0.292)	(0.86, 2, 0.650)	9.05, 2, 0.011	(0.74, 2, 0.735)	(1.82, 2, 0.403)	(1.34, 2, 0.513)
Sentence-initial latency (Model 4)	(2.70, 2, 0.100)	(1.70, 1, 0.192)	(2.51, 1, 0.113)	(0.08, 1, 0.776)	4.83, 1, 0.028	(2.21, 1, 0.137)

Values in parenthesis are for models that did not show statistically significant improvement in fit (*p* > .05). Values are for comparison with the previous best-fitting model, skipping over models that did not show statistically significant improvement in fit, except in the case of the general ability covariates which were included in all subsequent models. Model 1 was compared to an intercept-only model

**Table 6:** Factors predicting of text length, lexical diversity, spelling accuracy, and text quality. Standardized regression coefficients and 95% CI from best-fit models

	Length	Lexical diversity	Spelling errors	Thematic development	Organization	Overall quality
Composition fluency						
Mid-word latency	0.05 [− 0.21, 0.30]	–	0.03 [− 0.29, 0.34]	− 0.38 [− 0.63, − 0.12]**	–	− 0.13 [− 0.40, 0.13]
Word-initial latency	− 0.49 [− 0.76, − 0.23]***	–	− 0.12 [− 0.45, 0.21]	0.05 [− 0.22, 0.31]	–	− 0.12 [− 0.39, 0.15]
Mid-word disfluency	–	–	0.63 [0.14, 1.0]**	–	–	–
Word-initial disfluency	–	–	− 0.07 [− 0.50, 0.35]	–	–	–
Sentence-initial latency	–	–	–	–	− 20 [− 0.39, − 0.02]*	–
General ability						
Reading Comprehension	0.03 [− 0.16, 0.21]	0.17 [− 0.04, 0.38]	− 0.16 [− 0.35, 0.03]	0.25 [0.07, 0.44]**	0.25 [0.05, 0.45]**	0.27 [0.08, 0.46]**
Non-verbal reasoning	0.13 [− 0.05, 0.30]	− 0.09 [− 0.29, 0.11]	− 0.15 [− 0.32, 0.02]	− 0.07 [− 24, 0.10]	0.10 [− 0.08, 0.29]	0.13 [0.05, 0.31]

*p < 0.05, **p < 0.005, ***p < 0.001. Blank cells represent parameters that were absent in the best-fit model

General ability measures were, a priori*,* included in all models

General ability control measures were retained in all models. Reading comprehension was a significant predictor for each of the three text quality measures (thematic development, organization and overall quality). There were no other general ability effects. The effects of composition fluency on the final product were as follows.

Word-initial latency strongly predicted length, with students who spent longer preparing words (with longer interkey intervals prior to typing the word-initial character) producing shorter texts. Spelling accuracy was strongly predicted by within-word disfluency. We found no evidence of other effects of composition fluency on length, lexical diversity, or spelling accuracy.

We found two effects of composition fluency on compositional quality. Students’ whose typing was slower within words, tended to produce texts with weaker thematic development. Students who tended to hesitate for longer before starting a new sentence tended to produce text that had poorer organization. We found no evidence of other effects.

## Discussion

Our study aimed to test the frequently made claims (a) that difficulty with spelling retrieval and/or with the graphomotor skills necessary for inscription leads to slowed or disfluent production at the word level when composing spontaneous text and (b) that this production disfluency then disrupts the processing necessary for planning and structuring text content, and so results in a compositionally inferior final product. We found clear evidence in support of the first of these claims but, at best, weak evidence, for the second. We will discuss each in turn.

Composition fluency was predicted by independent measures of both typing fluency and spelling fluency. Within-word interkeystroke intervals when typing rehearsed sentences from memory (our typing fluency task) predicted within-word interkey intervals when composing. In fact, mean median within-word interkey intervals were only marginally longer during composition than during the typing fluency task. Similarly, word-initial intervals in the typing fluency task predicted word-initial intervals when composing, although in this case latencies were substantially greater when composing, consistent with the considerably greater lexical and orthographic retrieval demands of producing spontaneous text. These effects were after control for response time on the key-finding task, suggesting that ability to construct motor plans that string multiple keypresses together is to some extent distinct from being able to rapidly map letters onto single keys (see also Grabowski, 2008).

Key-finding response time also uniquely predicted within-word and word-initial latency when composing. More importantly from a process-disruption perspective, key-finding response time predicted within-word and word-initial pausing during composition: participants who were slower at mapping single letters onto keys were, when composing, more likely to hesitate for intervals of a length that might plausibly be associated with disruption of smooth flow from thought to screen.

Taken together, these findings are robust evidence that typing speed during composition is dependent, in part, on graphomotor skill. They indicate that higher level processes associated with developing content, syntax and spelling do not overdetermine rate of output when composing: keyboard knowledge and ability to string together sequences of keystrokes together explain considerable variance in measures of fluency when writers compose their own text. This finding is not surprising, and is consistent with and extends the findings of previous studies that found correlation between speed of written alphabet recall and composition fluency measures (Alves & Limpo, 2015; Limpo & Alves, 2017).

Previous research has failed to find evidence of a relationship between spelling ability, measured in terms of accuracy in a spelling-to-dictation task, and composition fluency in upper-primary students (Alves & Limpo, 2015; Graham et al., 1997). As we discussed in our introduction, however, spelling disfluency rather than spelling inaccuracy is the important factor when considering the possibility that lack of spelling ability might disrupt higher-level processing during composition. We found that fluency in written spelling-to-dictation predicted both word-level interkey interval and the probability of word-level disfluency. Response time on the spelling task predicted word-initial latency and the probability of potentially disruptive word-initial hesitation. Similarly, and interestingly, within-word interkey interval in the spelling task predicted within-word interkey interval when composing and strongly predicted the likelihood of within-word hesitation. It is likely that, once a word has been retrieved, fluent, “just-in-time” written production depends on the ability to then inscribe that word rapidly and effortlessly. The fact that, after control for typing fluency, within-word hesitation increased as a function of spelling fluency suggests that spelling ability affects composition time course and that, at least in a shallow orthography, these effects are not simply associated with word preparation but persist after output of the word has been initiated. This is consistent with findings from experimental written naming studies (Bertram et al., 2015; Scaltritti et al., 2016; Torrance et al., 2018) and studies of spelling to dictation (Rønneberg & Torrance, 2019), but has not, to our knowledge, previously been shown in spontaneous composition.

Our study therefore provided strong evidence that spelling and inscription fluency, measured independently and directly, and after statistical control for general ability, affects fluency when composing. In particular, lack of ability to spell and inscribe fluently increases the probability of pauses during composition of a duration that might, potentially, result from or in disruption to higher-level processing.

The second question to ask is whether or not these measures of composition fluency predict the quality of the resulting text. Our findings here were much less clear. As might be expected, participants who were less fluent composed shorter texts. Tendency to make spelling errors was associated with a greater tendency to hesitate within a word. The spelling effect is consistent with previous findings that general measures of typing speed during composition correlate negatively with the number of spelling errors in the third grade and seventh grade participants (Asker-Árnason et al., 2010; von Koss Torkildsen et al., 2016). Our finding may be due to either or both of writers hesitating within word to retrieve spelling or hesitating because they can see that they have made an error. Better understanding of possible mechanisms behind these effects are possible with more detailed analysis of where writers did and did not hesitate. These analyses are, however, beyond the present scope. The tendency to use a more lexically diverse vocabulary was not associated with a greater tendency to hesitate at the word level. Our results are similar to findings that lexical diversity measured as MTLD was not affected by disfluencies (Medimorec et al., 2017).^4^

We did not, however, find strong evidence of a relationship between word-level composition fluency and compositional quality (content and rhetoric) of the completed text. Of 12 possible effects, we found evidence for just one: participants who tended to type more slowly within-word tended to show weaker theme development—their texts tended toward weaker argumentation and tended to lack focus. An increase of 100 ms in median within-word latency (from a sample mean of 323 ms) was associated with a decrease of 0.32 in theme development (scored from 1 to 8). Note, however, that this effect was for within-word typing speed. We found no effects on any of the three quality measures for either of our two disfluency measures, and therefore no evidence that unusually long pauses disrupted higher-level processing.

We see, broadly, three possible explanations for failure in this and previous studies to find effects of processes disfluency on the compositional quality of the resulting text. It may be that there is potential for difficulties with spelling and inscription to interfere with upstream processes, but that in our present sample participants were sufficiently competent for this not to occur. We found clear evidence that children who were less fluent, relative to peers, when performing spelling and inscription tasks were also relatively less fluent when composing. The 25% of children who showed the most within-word disfluency paused for > 1 s before, on average, 1 in 18 keystrokes, and the 25% of children who paused most before words (with a 2 s threshold) did so before 1 in 9 words. It is possible, however, that this degree of hesitancy can be absorbed by the writing-production system without affecting composition quality. Alves and Limpo (2015) reported sixth grade writers composing a mean of 4.84 words for every pause in excess of 2 s (*SD* = 1.91), regardless of the location of pause within the text and found (weak) effects on text quality. Calculating the equivalent statistic for our sample gave *M* = 13.2 words (*SD* = 10.2). Part of this difference may be due to differences in recording methods. Alves and Limpo’s students wrote by hand, and times for in-air pen movements, between letters and between words, were included when determining pause durations. It is possible, however, that even given this, students in our sample were more fluent, although this is contrary to our expectations: typing, although practiced in Norwegian primary schools, remains the less preferred inscription modality for the majority of classrooms, including those that we sampled in this study.

Second, and more generally, it is possible that struggling with spelling retrieval and/or inscription does not, in fact, interfere with or divert attention from higher-level processes. It could be the case that motor planning or spelling retrieval, even when difficult, do not share processing resources or mechanisms with upstream processes. Third, it is possible that, even if delays at output result in information loss (the now-or-never bottleneck; Christiansen & Chater, 2016), this loss is recoverable. There is some support for this. Writing, unlike speech, provides an external record of what has just been said. Evidence from eye movement studies suggest that this might be used repeatedly to cue retrieval of what to say next (Torrance, Johansson, et al., 2016; Torrance, Rønneberg, et al., 2016), reducing demand on the memory buffers that are an essential part of a cascading text production system (van Galen, 1991). To our knowledge, the only experimental test of the hypothesis that increased inscription load reduces composition quality gave negative results: across two experiments in which adult competent typists were required to type with both hands and with one hand Medimorec and Risko (2016) found substantially slowed production in the one-hand condition. However, across a range of text measures they found limited evidence of effects on text quality. Where Medimorec and Risko did find effects in some cases, these pointed toward improved rather than reduced quality in the one-hand condition.

Our study failed to find evidence for a relationship between word-level composition fluency and text quality. This was despite strong evidence composition fluency was predicted by spelling and inscription ability. This is not, of course, to argue that spelling and inscription ability are unrelated to text quality in primary-aged children or persons with writing difficulties. The evidence reviewed by, for example, Feng et al (2019) points towards a clear association between transcription ability measures and the quality of their written composition. The findings that we present here do not, however, support the frequently made claim that these effects result from process disruption. A more parsimonious explanation for previous findings may simply be that the range of skills required to score well on tasks used to assess transcription ability (spelling to dictation, sentence copying, written alphabet recall), which include reading, short-term memory, and alphabet knowledge alongside orthographic knowledge and motor skill, correlates with the knowledge and skills required to produce well-structured and content-rich text.

In conclusion, therefore, we believe that the present study provides stronger evidence than has previously been available that in upper-primary-aged children the ability to spell and type fluently affects writing time course when composing full text. We did not, however, find strong evidence that fluency in composing affected the compositional quality of the completed text. This is inconsistent with claims made frequently in the existing literature that word-level disfluency when composing text disrupts the processing necessary for developing text content and structure. One possible reason for this is that although there was variation in fluency across our sample, children tended to have sufficient fluency in spelling and typing for this not to be disruptive to upstream processes. Future research could usefully reproduce the measures used in this study with a sample of children at an earlier developmental stage. In the meantime, however, we argue that the process-disruption hypothesis, as a claim that holds true across all writers, is less well supported than is typically assumed.

## Figures and Tables

**Fig. A2:**
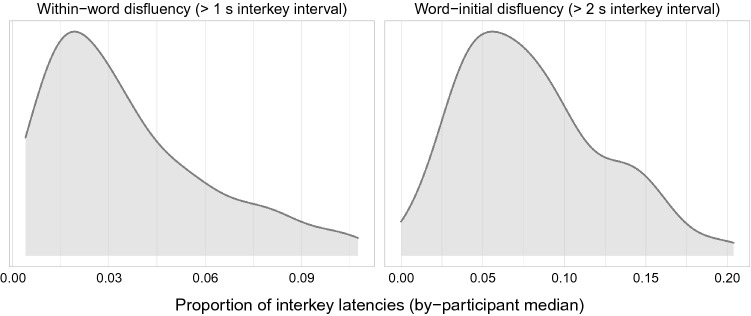
Distributions (Gaussian kernel density estimates) for by-subject medians of composition fluency measures

## Data Availability

Data will be made public via the Open Science Framework https://osf.io/7g5fx/. By-keystroke data are available on request.

## Appendix

Keystroke latency distributions for participants performing the composition task (i.e. composing multi-sentence texts).

See Figs. A1 and A2

## Footnotes

1 Here and throughout we use inscription, in preference to “handwriting and / or typing”, to refer to the motor planning and execution necessary to output retrieved word-spellings, we use handwriting and typing just when the specific output medium is relevant. Typing, in all cases, refers to computer keyboarding.

2 Mid-sentence is important here. Transcription processes are associated with word production. We deliberately exclude, therefore, time devoted to the conceptual and syntactic planning that is more likely to occur at the start of sentences.

3 Sentence- and word-boundaries are associated with more than one interkey-stroke interval. For words, for example, we might also consider the interval between pressing the key for the final character of the previous word and pressing the space bar. The interval between pressing the space bar and the first character of the new word is, however, where the effects of preparing the next word or sentence are most likely to be observed (e.g. Torrance et al., 2016a, 2016b; Wengelin, 2002).

4 Medimorec et al. (2017) did find a significant effect of composition disfluencies on type-token ratio even after controlling for length. We found an association between type-token ratio and word-initial disfluencies, with a tendency to use a broader vocabulary was associated with a tendency to hesitate before words. Type-token ratio however was negatively correlated with length, and the effect disappeared once we controlled for length.
